# Development of High-Grade Sarcoma After Second Dose of Moderna Vaccine

**DOI:** 10.7759/cureus.37612

**Published:** 2023-04-15

**Authors:** Edward Bae, Suhwoo Bae, Michael Vaysblat, Mohammed Abdelwahed, Kumar Sarkar, Stewart Bae

**Affiliations:** 1 Internal Medicine, State University of New York (SUNY) Downstate Health Sciences University, College of Medicine, Brooklyn, USA; 2 Internal Medicine, Donald and Barbara Zucker School of Medicine at Hofstra/Northwell, Manhasset, USA; 3 Pathology, Donald and Barbara Zucker School of Medicine at Hofstra/Northwell, Manhasset, USA; 4 Cardiology, Donald and Barbara Zucker School of Medicine at Hofstra/Northwell, Manhasset, USA; 5 Nephrology, Private Office, Flushing, USA

**Keywords:** moderna vaccine, sarcoma, high-grade sarcoma, coronavirus disease 2019, covid-19, adult internal medicine, outpatient medicine, musculoskeletal oncology, covid-19 vaccine

## Abstract

Coronavirus disease 2019 (COVID-19) is a disease characterized by predominantly respiratory symptoms, which can progress to respiratory failure. Due to the novelty of the vaccines, it is difficult to assess if there are any associated long-term side effects. Here, we present a case of an elderly female who received the Moderna COVID-19 vaccine and developed a high-grade sarcoma at the site of the injection. A 73-year-old female with a past medical history of hypertension, hyperlipidemia, and renal angiomyolipoma status post resection in 2019 presented with worsening right upper arm swelling for the past two weeks. She noticed the swelling two to four days after receiving her second dose of the Moderna vaccine within 1 cm from the prior injection site. Physical examination was remarkable for a 6 cm, circular, mobile, soft mass present in the right upper arm. MRI with and without contrast revealed a 5.2 cm soft tissue mass overlying the triceps region with irregular features concerning for malignancy. Fine needle aspiration revealed pathologic characteristics indicative of high-grade sarcoma. The patient ultimately had resection of the mass four months after the initial visit and was diagnosed as having grade 3, stage IIIA undifferentiated, pleomorphic high-grade sarcoma. Herein, we present a case demonstrating the development of high-grade sarcoma at the injection site in an elderly female patient within days of receiving the second dose of the Moderna COVID-19 vaccine. Currently, it is unclear whether there is a true association between the vaccines and malignancy or inflammatory response exacerbating underlying malignancy. This case highlights the necessity to investigate and be aware of such rare, adverse complications that may be associated with the novel COVID-19 vaccinations to guide physicians in their differential diagnosis.

## Introduction

Coronavirus disease 2019 (COVID-19) is a disease characterized by predominantly respiratory symptoms that can progress to respiratory failure. Given the mortality and morbidity associated with COVID-19, novel COVID-19 vaccines were invented in response. Due to the novelty of the vaccines and the innovative mRNA technology the vaccinations utilize, it is difficult to assess if there are any long-term side effects associated with vaccination. Current literature reports headache, fever, fatigue, chills, and tenderness at the injection site as the most common short-term side effects [1]. In addition, other studies report anaphylactic reactions, myocarditis, and thromboembolic events as severe but rare complications as well [2-4]. Based on our exhaustive search, there is no reported association between COVID-19 vaccines and malignancy. Here, we present a case of an elderly female who received the Moderna mRNA-1273 COVID-19 vaccine and developed a high-grade sarcoma at the site of the injection.

## Case presentation

A 73-year-old female with a past medical history of hypertension, hyperlipidemia, and renal angiomyolipoma status post resection in 2019 presented with worsening right upper arm swelling for the past two weeks. She noticed the initial swelling two to four days after receiving her second dose of the Moderna vaccine within 1 cm from the prior injection site. This was initially attributed to phlebitis. She reported mild, non-radiating pain on palpation. Vitals were unremarkable and the patient was afebrile. Physical examination was remarkable for a 6 cm, circular, mobile, soft mass present in the right upper arm without fluctuance, erythema, or warmth. She had no neurological deficits and radial pulses were present.

**Figure 1:**
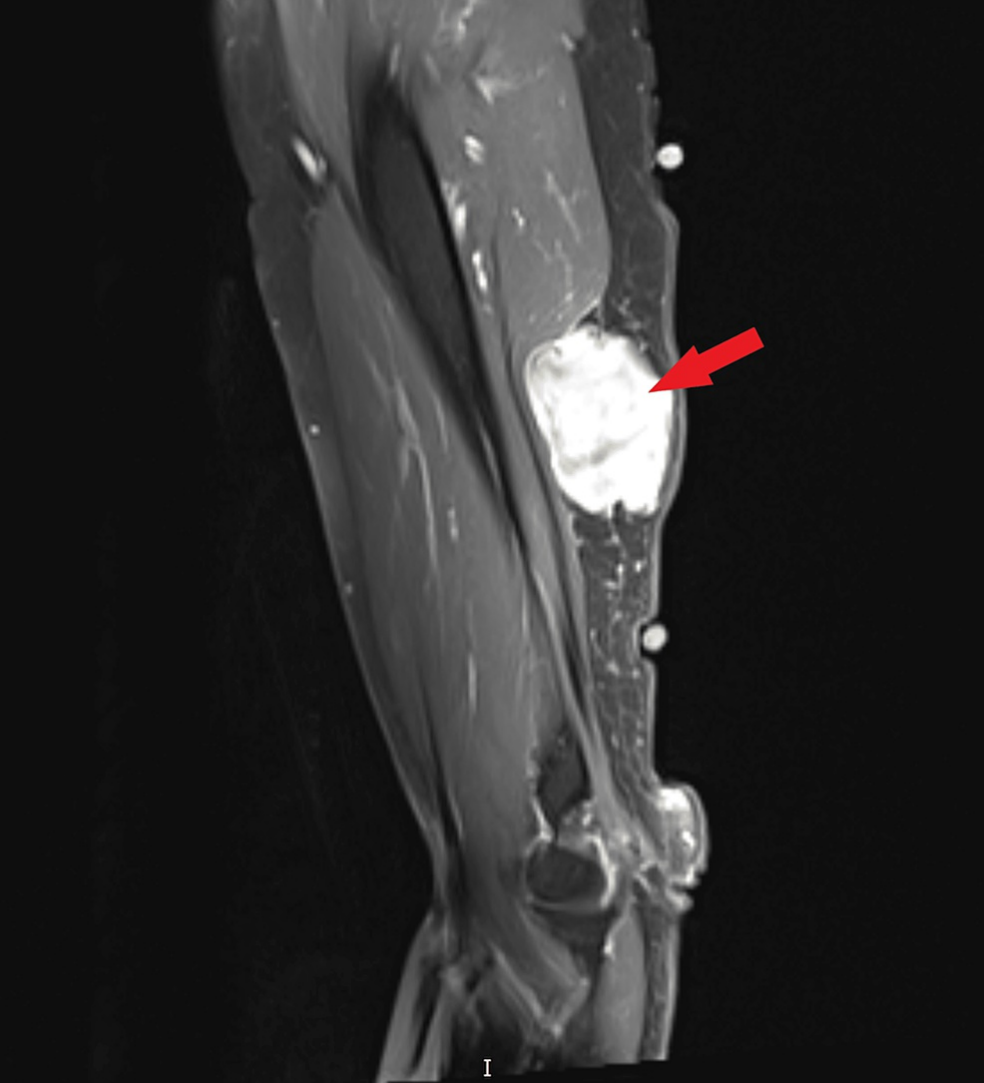
MRI of the humerus showing 5.2 cm soft tissue mass (red arrow) within the subcutaneous fat overlying the triceps region concerning for malignancy.

Complete blood count (CBC) was unremarkable with no leukocytosis, leukocytopenia, or anemia. The comprehensive metabolic panel (CMP) was unremarkable. The patient was advised to obtain an MRI with and without contrast, which revealed a 5.2 cm soft tissue mass within the subcutaneous fat, overlying the triceps region, with irregular features concerning for malignancy (Figure 1). A subsequent ultrasound-guided core biopsy with fine needle aspiration (FNA) was performed.

**Figure 2:**
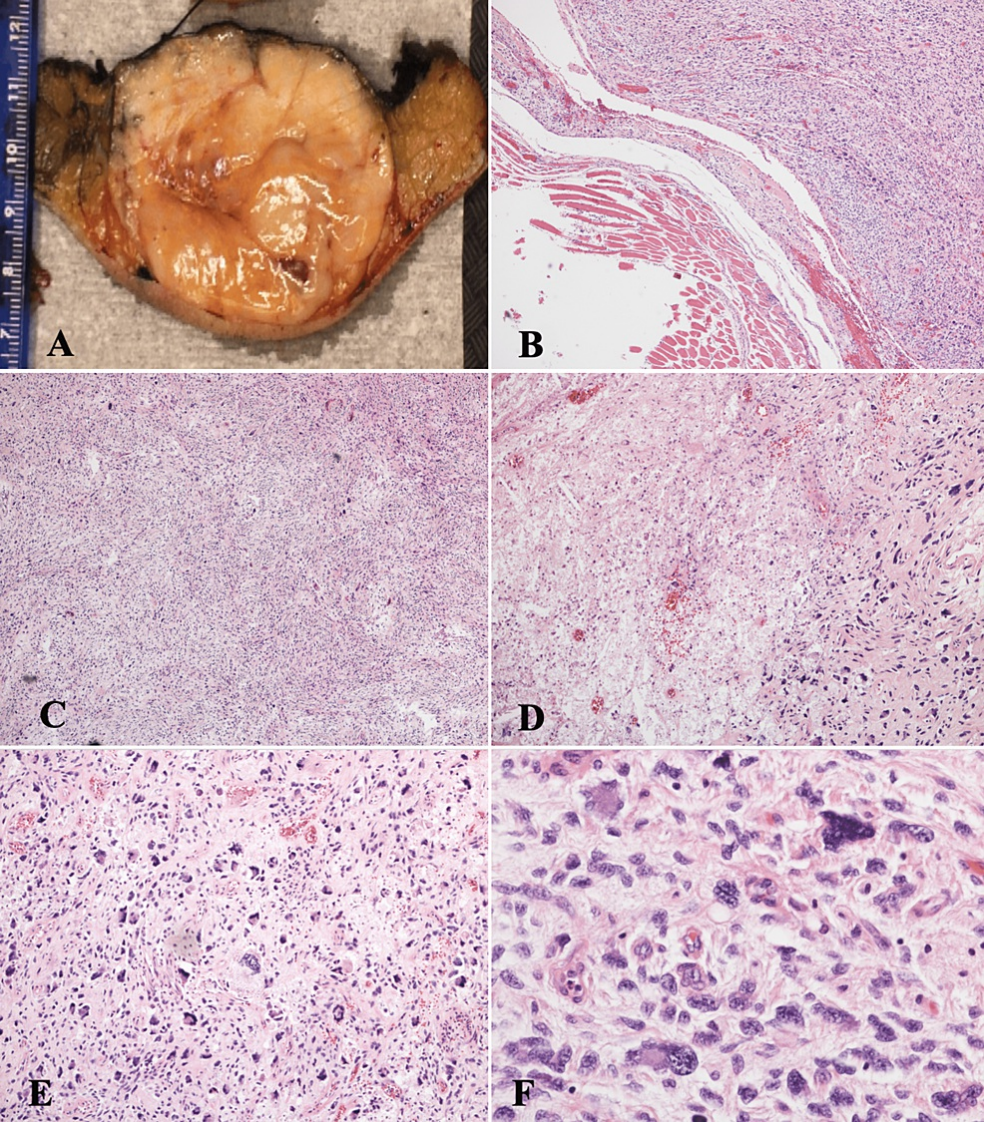
Right arm mass resection. (A) Gross picture, 8.8 x 7.3 x 5.0 cm soft tan-pink well-defined mass. (B) Centrally located bulging edge of the soft tissue mass surrounding normal skeletal muscle (hematoxylin and eosin (H&E) stain, x4). (C) Center of the mass showing intersecting spindle cells with focal necrosis and high-grade atypia (H&E stain, x4). (D) Center of the mass showing high-grade sarcoma composed of pleomorphic spindle cells and numerous tumor giant cells with central necrosis (H&E stain, x10). (E) High-grade sarcoma showing highly atypical nuclei with 15 mitoses per 10 high power fields (HPF) including abnormal forms (H&E stain, x10). (F) High-grade pleomorphic sarcoma showing highly atypical nuclear features (H&E stain, x40).

FNA revealed a hypercellular specimen consisting of bland spindle cells mixed with anaplastic cells, with multinucleation, pleomorphism, hyperchromatic, eccentrically located nuclei with eosinophilic cytoplasm, and brisk atypical mitotic features. Based on the examination, imaging, and FNA results, the patient underwent surgical resection with surgical oncology four months after the initial visit. At that point in time, the patient’s sarcoma was estimated to be 20 x 15 cm on physical examination. The patient was scheduled with surgical oncology for resection of the mass and had removal with clean margins. No spread to regional lymph nodes was evident. Grossly, the mass was 8.8 x 7.3 x 5.0 cm, tan-pink, well-defined, and soft, with a centrally located, bulging area measuring 2.3 cm from the nearest margin. The cut surface of the mass was tan-white to pale-pink, somewhat fleshy, lobulated, focally hemorrhagic, and rubbery-to-firm in consistency (Figure 2A). Microscopically, the soft tissue mass was abutting unremarkable skeletal muscle (Figure 2B). The center of the mass showed high-grade sarcoma composed of pleomorphic spindle cells and numerous tumor giant cells with central necrosis (more than 30%) (Figures 2C, 2D). Nuclei were highly atypical (G3) and had 15 mitoses per 10 high power fields (HPF), including abnormal forms (Figure 2E). There was no lymphovascular invasion and all margins were free of sarcoma. Tumor cells were negative for AE1/3, Cam 5.2, S-100, SOX-10, HMB-45, smooth muscle actin (SMA), desmin, and myogenin. CD34 showed a nonspecific stain (Figure 2F). Given the cytomorphology and immunophenotypic features, the patient was diagnosed as having grade 3, stage IIIA undifferentiated, pleomorphic high-grade sarcoma. She was then started on adjuvant radiation therapy after surgery with full recovery and no recurrence to date.

## Discussion

Undifferentiated pleomorphic sarcoma (UPS) was first described in the literature back in the early 60s as a form of undifferentiated fibroblasts formed in the background of histiocytic growth [5]. Later, it was divided into five categories: storiform-pleomorphic, myxoid (myxofibrosarcoma), giant cell (malignant giant cell tumor of soft parts), inflammatory, and angiomatoid. It was described in different organs including the skin, heart, thyroid, colon, breast, and muscle [5]. The pathogenesis of UPS has been a source of debate for the last three decades [5]. While some investigators linked it to mesenchymal stem cells, other investigators linked it to multiple incidents of genetic alterations, resulting in the activation of oncogenes or inactivation of tumor suppressor genes [5]. Some authors linked UPS with radiotherapy, especially in the head and neck. Other genetic pathways may also be implicated in UPS tumor biology, including vestigial-like family member 3 (VGLL3), Dickkopf-related protein 1 (DKK1), tumor protein 53 (TP53), cyclin-dependent kinase inhibitor 2A (CDKN2A), retinoblastoma-associated protein (RB1), and transcriptional regulator ATRX genes [5]. Differential diagnosis of UPS includes other dedifferentiated/undifferentiated soft tissue tumors, including pleomorphic liposarcoma, pleomorphic leiomyosarcoma, pleomorphic rhabdomyosarcoma, inflammatory malignant fibrous histiocytoma, and myxofibrosarcoma [6]. UPS is an aggressive tumor with a high recurrence rate, with higher recurrence rates in radiation-associated UPS than sporadic. In a current retrospective study, recurrences and metastases occurred in 14.1% and 7.8% of the cases, respectively. Early disease recognition and an adequate treatment strategy are the most important interventions to improve overall prognosis. The five- and 10-year overall survival rates were 60% and 48%, respectively [7].

Based on an extensive search, we describe the first case of rapidly progressive, high-grade undifferentiated sarcoma that seems to have a strong association with the Moderna vaccination. Although the literature well documents the efficacy of the vaccines in immunocompromised patients such as those with malignancy [8], there are currently no studies reporting any association between the development of sarcoma or worsening of malignancy and the COVID-19 vaccinations as a potential cause.

It is well established that other vaccines may lead to the formation of subcutaneous nodules, particularly in children after the administration of the pertussis vaccine [9]. A review of the literature shows a recent case report that describes the development of a soft tissue mass shortly after the administration of a COVID-19 vaccine. A soft tissue growth with malignant characteristics was found to be developed shortly after administration of the COVID-19 Moderna vaccine with MRI. Pathological analysis subsequently demonstrated non-necrotizing granulomatous inflammation without evidence of neoplasia. This case reported the development of a localized granulomatous reaction that presented as a mass following the COVID-19 Moderna booster vaccine that resolved spontaneously without any medical intervention [10].

Although a non-malignant growth has been reported as a rare complication of the Moderna COVID-19 vaccine, it is important to consider other unique complications that may be implicated. It is well-documented in the literature for over 20 years that high-grade sarcomas have been linked to vaccine administration in felines [11]. Although these occurrences are uncommon, the development of such tumors is found to be iatrogenic [11]. Vaccination against rabies and feline leukemia virus is thought to be the most common inciting cause although the mechanism of action is poorly understood [11]. This study has also shown that the injection site sarcoma in these animals is extremely locally invasive and recurrence is common even with aggressive treatment [11]. More recently, case reports have indicated the development of high-grade sarcoma in canines as well, although rare when compared to feline cases [12].

Our patient has a previous history of renal angiomyolipoma. Renal angiomyolipoma is often associated with tuberous sclerosis complex (TSC), which often presents with a variety of complications such as subependymal nodules, subependymal giant cell astrocytomas, skin lesions such as facial angiofibroma, renal cell carcinoma, cardiac rhabdomyoma, and so forth [13]. Although our patient had no clinical indication or history of TSC, it is unknown whether renal angiomyolipoma in itself can increase the risk of other malignancies. One study has reported cases of renal angiomyolipoma with rare malignant transformation into adenocarcinoma and sarcoma in the same kidney even months after partial nephrectomy [14]. Renal angiomyolipoma is often viewed as a benign mesenchymal tumor but these cases have shown rare cases of malignant transformation. It may be prudent to investigate whether renal angiomyolipoma in itself can pose a risk factor for the development of malignant high-grade sarcoma in other locations.

Interestingly, another recent case report has reported spontaneous tumor regression following the administration of the Moderna COVID-19 vaccine [15]. In this case, the patient was a 61-year-old female diagnosed with T2N0MX metastatic myoepithelial carcinoma of the left parotid gland [15]. After parotid resection and postoperative radiation therapy completion, metastatic disease in the lung was evident [15]. In this case, the decision was made to follow with close surveillance due to the lack of symptoms and standardized therapy for myoepithelial carcinoma [15]. Follow-up CT scans demonstrated 50%, 67%, and 73% reduction at three, six, and nine months, respectively, after the second dose of the vaccine [15]. It was found that between the prevaccination and postvaccination lung biopsy specimens, there was an increase in CD8+ T cell, CD4+ T cell, B cell, natural killer cell, and dendritic cell infiltration into the tumor postvaccination [15]. The authors hypothesized that the vaccine may have stimulated the immune system and led to a vigorous anticancer response [15]. This poses the question of whether the Moderna COVID-19 vaccine may have different outcomes or complications depending on the exact histopathology of malignancy and oncological history of the patient, as well as the individual unique response of the innate immune system.

## Conclusions

Herein, we present a case demonstrating the development of undifferentiated, pleomorphic high-grade sarcoma at the injection site in an elderly female patient within days of receiving the second dose of the Moderna COVID-19 vaccine. Currently, it is unclear whether there is a true association between novel vaccinations and the development of malignancy. A review of the literature does not show any other case reports demonstrating malignancy after receiving the Moderna vaccine. This should be further investigated to see if there is an association and, if so, the mechanism thereof. Despite the uncertainty, it is necessary to note such cases of potentially rare, adverse complications as clinicians should consider the side effects of vaccinations in their differential diagnosis to guide patients in shared decision-making. Early recognition of the disease, as in this patient, proved to be vital for her quality of life and prevented further progression.
